# The complete mitochondria genome sequence of *Proceras venosatum* (Walker)

**DOI:** 10.1080/23802359.2018.1532333

**Published:** 2018-10-31

**Authors:** Shiqiang Xu, Gan Cao, Jihua Wang

**Affiliations:** a Guangdong Key Laboratory for Crops Genetic Improvement, Crops Research Institute, Guangdong Academy of Agricultural Sciences, Guangzhou, China;; b State Key Lab for Conservation and Utilization of Subtropical Agric-Biological Resources, Guangxi University, Nanning, China

**Keywords:** *Proceras venosatum*, mitochondria genome, phylogenetic relationship

## Abstract

*Proceras venosatum* (Walker) is one of the major pests, which caused yield losses of sugarcane in the world. The complete mitochondria genome (mtDNA) is reported; a circular molecule of 15,378 bp in size, which including 39.69% for A, 11.89% for C, 7.19% for G, and 40.96% for T. There are 36 genes in the mtDNA, including 3 species with 15 protein-coding genes, 2 different species ribosomal RNA genes (S and L rRNA species), 22 transfer RNA genes (20 RNA species). *Proceras venosatum* (Walker) and other 18 species belonging to lepidopteran were carried out phylogenetic analyses by used MEGA 6.06 with Neighbor-Joining methods. The mtDNA of *P. venosatum* (Walker) were clustered in lepidopteran superfamilies.

*Proceras venosatum* (Walker) (Chilo sacchariphagus Bojer) belonging to Lepidoptera, is one kind of sugarcane stem borers, which is the major pest causing more than 7% of sugarcane yield losses per year in China and induces diseases (Weng et al. 2006; Lao et al. 2014). Lepidoptera is one of the largest insect orders, most of them are pests, but the phylogenetic relationships have yet to be clearly described (Kim et al. 2011). The mtDNA was the easiest to isolate and characterize, and conservation in gene content, homology among genes could be established readily, which are available for 4 lepidopteran superfamilies: Tortricoidea (Adoxophyes); Pyraloidea (Ostrinia); Bombycoidea (Bombyx, Antheraea), and Papilionoidea (Coreana) (Brown et al. 1994; Cameron and Whiting 2008). In this study, the complete genome of *Proceras venosatum* was sequenced and constructed phylogenies based on phylogenetic with Neighbor-Joining method.

*Proceras venosatum* were collected from sugarcane planted in Baiyun farm of Guangdong Academy of Agricultural Sciences, Guangdong province. The heads of *P. venosatum* collected in Zhongluotang (N23°23′21.48″,E113°25′55.43″) were treated for total genomic DNA isolated and stored in Guangdong Key Laboratory for Crops Genetic Improvement, Guangzhou. The mtDNA of *P. venosatum* was sequenced with Illumina Hiseq 2500 System and assembled with *P. massoniana* (Genebank accession No: KC427272) as a reference by used MITObim v1.8 (Hahn et al. 2013). The cpDNA annotated the genes by GENEIOUS R8 (Biomatters Ltd., Auckland, New Zealand) and the gene map (Figure S1) was drawn via the internet tool OGDRAW v1.2 (Lohse et al. 2013；Lohse et al. 2007) (http://ogdraw.mpimp-golm.mpg.de/).

The circular cpDNA of *P. venosatum* (Genebank accession No: KU188518) is 15,378 bp in length, including 39.69% for A, 11.89% for C, 7.19% for G, and 40.96% for T. There are 36 genes identified in the mitochondrion, including 14 protein-coding genes, 2 ribosomal RNA genes (2 rRNA species), 22 transfer RNA genes (20 tRNA species). There are 15 genes (ND2, COX1, COX2, ATP8, ATP6, COX3, ND3, ND5, ND4, ND4L, ND6, CYTB, ND1, S-rRNA, and L-rRNA), while not occurred in other genes. Most genes were single copy, while 2 tRNA species (Leu, Ser) occurred in double copies.

Phylogenetic analysis was constructed, which was based on the 15 protein-coding gene sequences of *P. venosatum* and other 18 species belonging to lepidopteran by MEGA 6.06 (Tamura et al. 2013). The phylogenetic tree based on Neighbor-Joining method (NJ) demonstrated that *P. venosatum* was clustered in Pryalidae (Figure 1).

**Figure 1.**
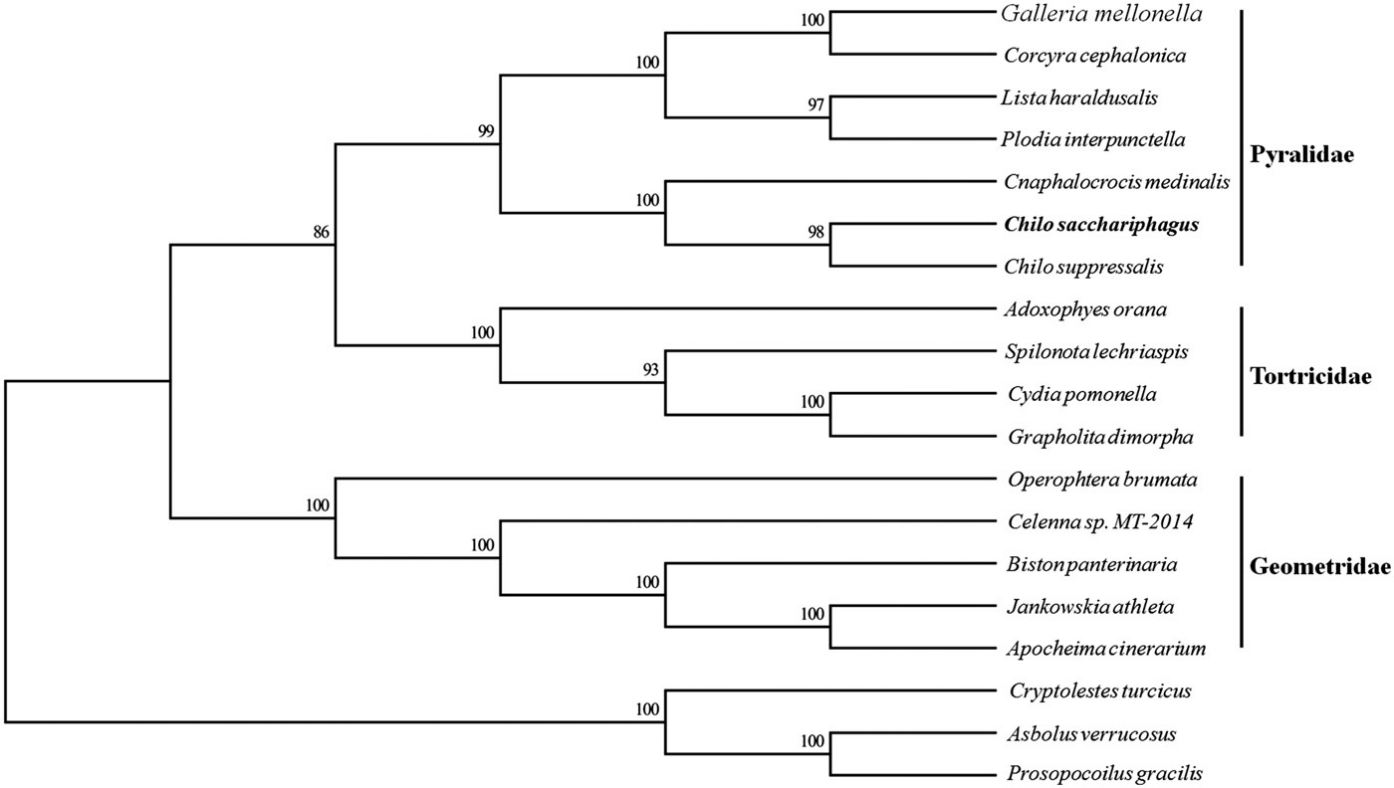
The ML, the phylogenetic tree of the *Proceras venosatum* and related families based on genome sequence. Numbers labeled beside the node are bootstrap support values. *Proceras venosatum* and *Chilo suppressalis* were the most similar.
